# A Novel Lateral Approach to the Assessment of Vocal Cord Movement by Ultrasonography

**DOI:** 10.1007/s00268-017-4151-z

**Published:** 2017-07-27

**Authors:** Takahiro Fukuhara, Ryohei Donishi, Eriko Matsuda, Satoshi Koyama, Kazunori Fujiwara, Hiromi Takeuchi

**Affiliations:** Department of Otolaryngology, Head and Neck Surgery, Tottori University Faculty of Medicine, 36-1 Nishicho, Yonago, 683-8504 Japan

## Abstract

**Background:**

Ultrasonography is a non-invasive technique that is commonly used by endocrinologists and endocrine surgeons to examine the thyroid region and could be useful for the assessment of vocal cord movement by these specialists. However, previous studies reported a low rate of successful visualization of vocal cord movement by ultrasonography. To address this issue, we devised a novel ultrasonographic procedure for assessing vocal cord movement indirectly by observing the arytenoid movement from a lateral view.

**Methods:**

Subjects were 188 individuals, including 23 patients with vocal cord paralysis and 13 with vocal cord paresis. We performed ultrasonographic assessment of vocal cord movement using two different procedures: the conventional middle transverse procedure and the novel lateral vertical procedure.

**Results:**

The rate of visualization of vocal cords with the middle transverse procedure was 70.2% and that of the arytenoid cartilage with the lateral vertical procedure was 98.4%. The lateral vertical procedure enabled visualization of all patients with vocal cord paresis/paralysis and detected all 23 patients with vocal paralysis; only one of 13 patients with vocal cord paresis was positively identified. The conventional procedure enabled visualization of 21 of 36 patients with vocal cord paresis/paralysis with high accuracy. There was no false-positive case in either procedure.

**Conclusion:**

The proposed lateral vertical procedure improved the rate of visualization of vocal cord movement by ultrasonography, suggesting that it is a useful technique to screen for vocal cord paralysis by ultrasonography.

**Electronic supplementary material:**

The online version of this article (doi:10.1007/s00268-017-4151-z) contains supplementary material, which is available to authorized users.

## Introduction

Vocal cord paralysis caused by recurrent nerve palsy is a major complication of thyroid surgery [1–3]. Therefore, it is important for endocrinologists and endocrine surgeons to assess vocal cord movement. The gold standard procedure for assessing vocal cord movement is flexible nasopharyngoscopy. However, nasopharyngoscopy is partly invasive [4] and not commonly performed by endocrinologists or endocrine surgeons [5]. On the other hand, ultrasonography is non-invasive and is commonly used by these specialists for surveys of the neck [6]. Therefore, ultrasonography would be a useful tool for the assessment of vocal cord movement by these clinicians.

Several studies have described ultrasonographic assessment of vocal cord paralysis. However, the rate of successful visualization of vocal cord movement by ultrasonography is lower than that achieved by nasopharyngoscopy; consequently, ultrasonography is not considered an adequate alternative tool [7]. The visualization rate is reported to be approximately 50% [8]. The low visualization rate was attributed to the calcification or angle of the thyroid cartilage [7, 9]. In addition, previous studies used procedures in which they directly observed the vocal cord surrounded by air [8, 10–14].

We devised a novel ultrasonographic procedure for assessing vocal cord movement by observing the arytenoid movement from a lateral view. This novel procedure is based on the indirect assessment of vocal cord movement by observing the movement of the arytenoid cartilage through the shortest distance. The arytenoids are surrounded by tissues without air and can therefore be observed easily, whereas the true vocal cords are surrounded by air. Therefore, this novel procedure is expected to improve the visualization rate for assessing vocal cord movement. This study verified the usefulness of the proposed ultrasonographic procedure for assessing vocal cord movement.

## Patients and methods

### Patients

Vocal cord movement was assessed in patients who received cervical ultrasonography for screening or further evaluation (Table 1) and who underwent nasopharyngoscopy within 6 months of ultrasonography. A total of 188 consecutive patients were enrolled between September 2013 and April 2014. The study was approved by the Institutional Review Board of Tottori University and was performed in accordance with the Declaration of Helsinki.

**Table 1:** Clinical characteristics of the subjects

Total number		188
Gender	Male	89
	Female	99
Age (average)		63.4 ± 15.1
Vocal palsy on laryngoscopy	No	152
	Paresis	13
	Paralysis	23
Gender of the patients with vocal palsy	Male	21
	Female	15
Age of the patients with vocal palsy (average)		70.5 ± 9.8
Primary disease	Thyroid disease	
	Malignant tumor	52
	Benign tumor	10
	Adenomatous goiter	32
	Autoimmune thyroiditis	6
	Parathyroid tumor	5
	Head and neck tumor	
	Malignant tumor	42
	Benign tumor	2
	Cyst	4
	Lymph node	
	Malignant Lymphoma	2
	Lymphadenitis	11
	Globus	10
	Others	12

### Measurement procedures

We used an ACUSON S2000 ultrasound system with a 9-MHz linear transducer (Siemens Medical Solutions USA). We performed ultrasonographic assessment of vocal cord movement using two different procedures in conjunction with cervical ultrasonographic examination.

**Fig. 1:**
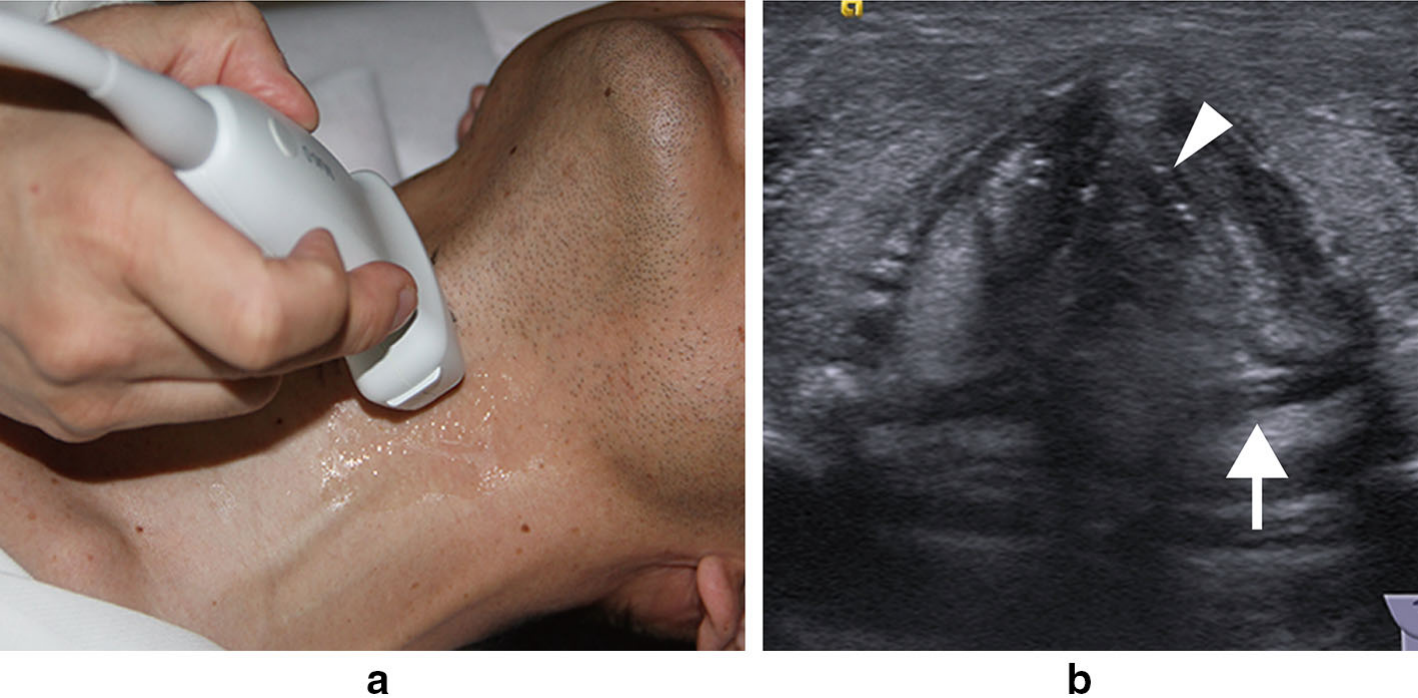
**a** Probe placed transversely over the middle portion of the thyroid cartilage. **b** Ultrasonographic view showing a normal visualized case. True vocal cord (*arrowhead*); arytenoid cartilage (*arrow*)

**Fig. 2:**
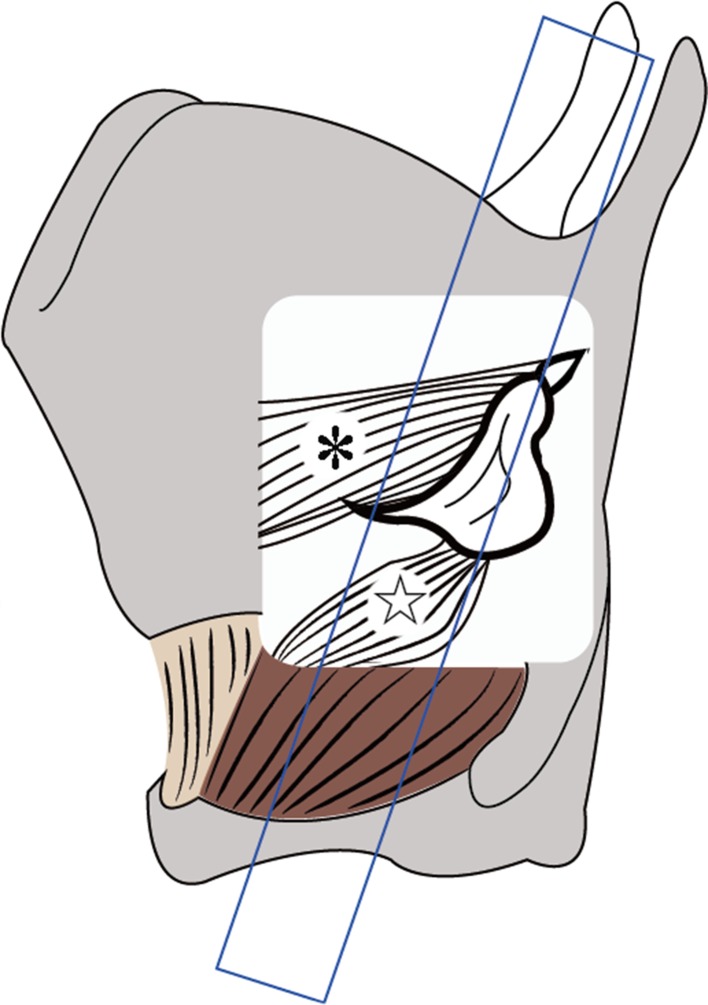
Inner structures of the larynx visualized through the window of larynx by ultrasonography with the lateral procedure. Arytenoid cartilage (*arrow*); *, thyroarytenoid muscle; ☆, lateral cricoarytenoid muscle

**Fig. 3:**
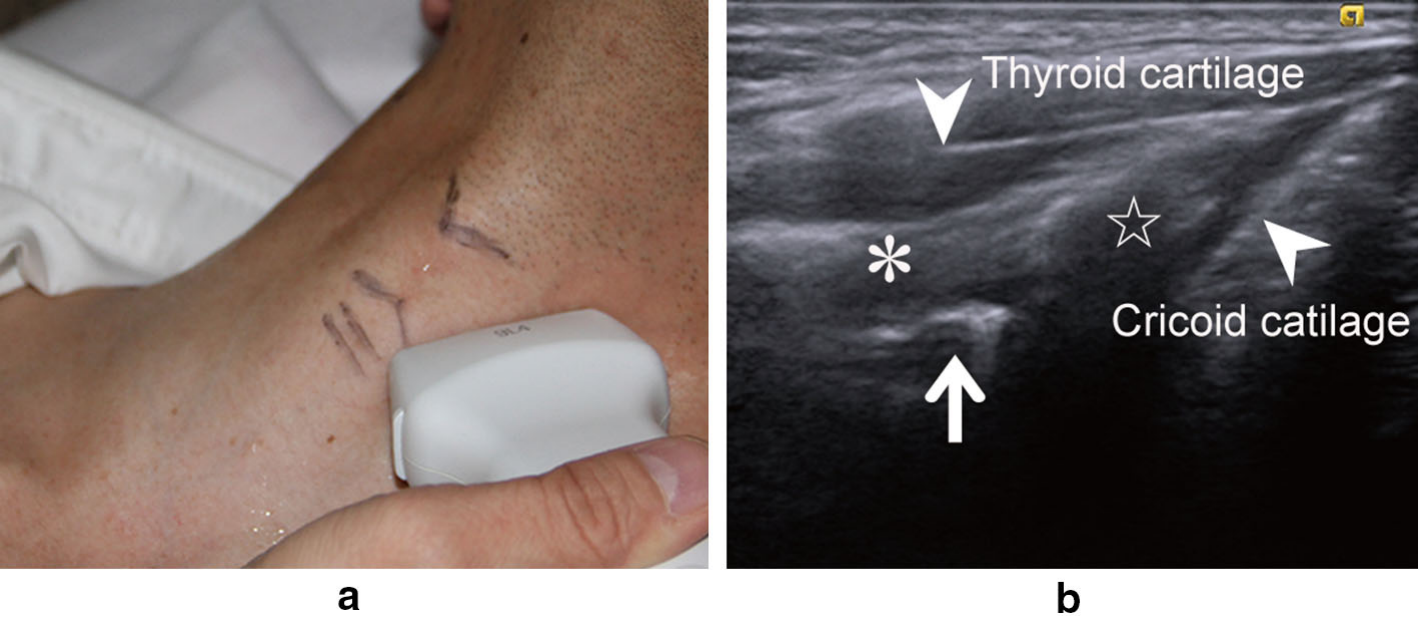
**a** Probe placed vertically along the left lamina of the thyroid cartilage. **b** Ultrasonographic view of the lateral vertical procedure. Muscular process of the arytenoid cartilage (*arrow*); *, thyroarytenoid muscle; ☆, lateral cricoarytenoid muscle

The patients underwent cervical ultrasonographic examination in the supine position with their neck slightly extended and at the end of the examination underwent ultrasonographic assessment of vocal cord movement by two different procedures in the same position. In one of the procedures, we placed the linear transducer transversely over the middle portion of the thyroid cartilage and observed the bilateral vocal cords bounded by air (Fig. 1). In the other procedure, the transducer was placed vertically at 1–1.5 cm inside and parallel to the lateral border, along the oblique line of the thyroid cartilage because the arytenoid cartilage is located behind the oblique line of the thyroid cartilage (Fig. 2). The transducer was placed at the right and left sides of the thyroid cartilage lamina, and the movement of the right and left arytenoid cartilages was observed separately (Fig. 3).

**Fig. 4:**
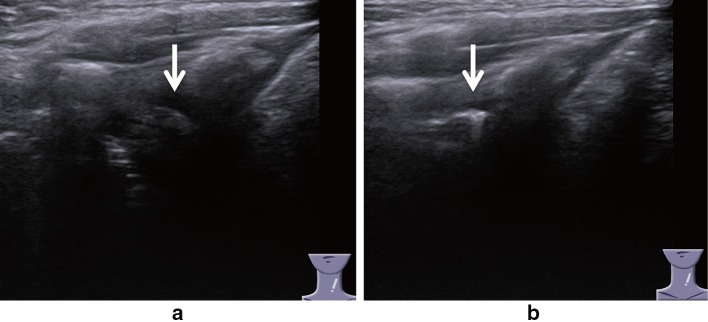
Images showing movement of the muscular process of the arytenoid cartilage (MP), visualized by ultrasonography with the lateral vertical procedure. *Arrows* show the MP. **a** Abduction. The MP is not visible. **b** Adduction. The MP is visible

In our hospital, otolaryngologists classify vocal cord movements into three groups based on the results of nasopharyngoscopy: normal, paresis, and paralysis. However, in ultrasonography, bilateral true vocal cord visualization is considered an indicator of the successful performance of the middle transverse procedure, whereas arytenoid visualization indicates a successful lateral vertical procedure. The criterion for the positive detection of vocal cord paresis/paralysis using the middle transverse procedure was asymmetric abduction and adduction movements of the true vocal cords. However, simultaneous observation of the right and left sides was not possible in the lateral vertical procedure, and we were unable to compare ultrasound images between the two sides. Therefore, the positive diagnostic criterion for vocal cord paralysis in the lateral vertical procedure was fixation of the arytenoid cartilage to avoid subjectivity (Fig. 4).

First, the percentages of visible true vocal cords or arytenoid cartilages were compared between the two procedures. Then, the results of the evaluation of vocal cord movement by ultrasonography were confirmed for each procedure based on the results of nasopharyngoscopy.

### Statistical analysis

Statistical analyses were performed using SPSS software (IBM SPSS Statistics 22; IBM, Japan). The percentages of visible true vocal cords or arytenoid cartilages were compared between the two procedures using the McNemar test.

## Results

The characteristics of the 188 subjects, including 23 patients with vocal cord paralysis and 13 patients with vocal cord paresis, are shown in Table 2.

**Table 2:** Results of ultrasonographic evaluation of vocal cord movement with each procedure

		Normal	Paresis	Paralysis
Conventional middle transverse procedure	Non-assessable	41	6	9
	Positive	0	6	14
	Negative	111	1	0
	Total	152	13	23
Novel lateral vertical procedure	Non-assessable	3	0	0
	Positive	0	1	23
	Negative	149	12	0
	Total	152	13	23

The visualization rate of vocal cords with the middle transverse procedure was 70.2% (assessable, 132; non-assessable, 56), and the visualization rate of arytenoid cartilage with the lateral vertical procedure was 98.4% (assessable, 185; non-assessable, 3). In fact, arytenoid cartilages were not visualized in only three patients. The visualization rate was significantly higher with the lateral vertical procedure than with the middle transverse procedure (*P* < 0.001).

The results of the evaluation of vocal cord movement by ultrasonography with each procedure were compared with those obtained by nasopharyngoscopy (Table 2). In each group, some cases were not assessable with the middle transverse procedure; however, for the visualized cases, this procedure was highly accurate. On the other hand, the accuracy of evaluation of vocal cord paresis by the novel lateral vertical procedure was low, and all of the false-negative cases involved vocal cord paresis. There were no false-positive cases in either of the procedures.

## Discussion

It is important for endocrinologists and endocrine surgeons to determine whether the vocal cords are paralyzed. The gold standard for assessing vocal cord paralysis is nasopharyngoscopy; however, this technique is ordinarily used by otolaryngologists. On the other hand, ultrasonography is commonly used by endocrinologists and endocrine surgeons to examine the thyroid region [6]. Therefore, assessment of vocal cord movement by ultrasonography would be efficient and economical [15]. Several studies over the past two decades have described the use of ultrasonography to assess the vocal cords [16, 17]. Initially, ultrasonography was not sufficient for clinically useful observation. Owing to technological innovations; however, it has recently become easy to visualize the vocal folds from the body surface [18]. However, the success rates of observing vocal cord movement in these studies varied widely, and ultrasonography is still not thought to be a sufficiently precise tool to assess vocal cord movement [8, 10–14].

**Fig. 5:**
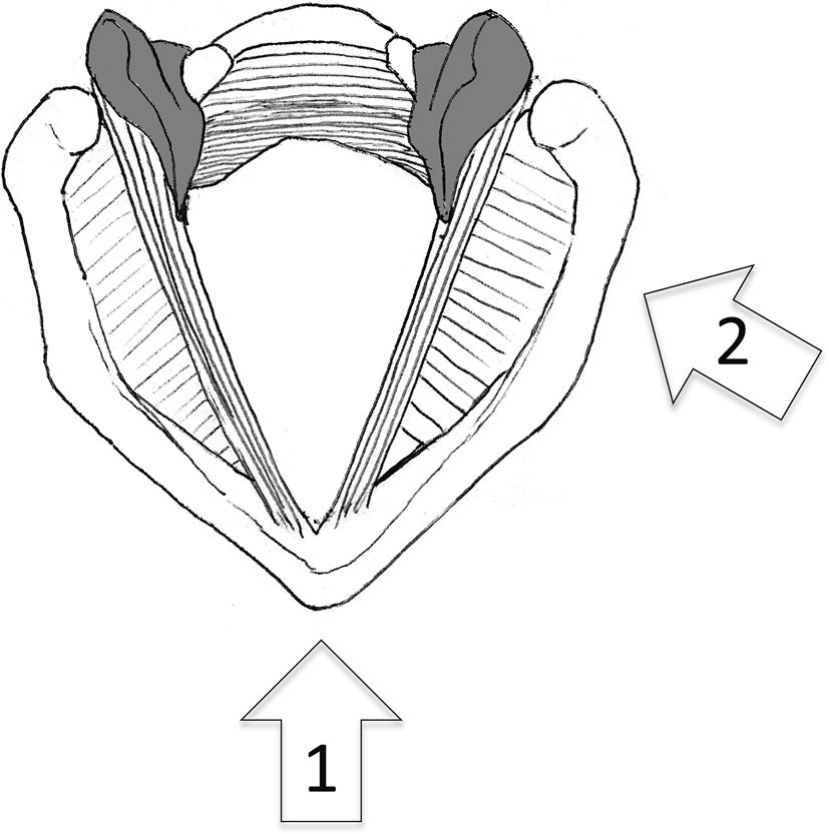
Arytenoid cartilages are colored in *gray*. * 1* The middle approach. * 2* The lateral approach

These previous reports shared a common feature, namely that they observed the vocal cords directly by the middle transverse procedure. However, this procedure has the notable disadvantage that it is easily influenced by the calcification or angle of the thyroid cartilage [7, 9]. Additionally, we suspected that the main factor responsible for the low rate of successful visualization of vocal cords is the poor ultrasonic permeability of air (Fig. 5).

Many past studies using the middle transverse procedure that defined success as bilateral true vocal cord visualization reported visualization rates of 70–87% [10–14]. The variation in this rate may have been caused by differences in the proportion of women, because visualization rates were much higher in women than in men [10–14]. In this study, the true vocal cords were the targets of observation with the middle transverse procedure, because the true vocal cords directly reflect the function of the recurrent nerve. Our visualization rate with the middle transverse procedure was similar to that reported in previous studies.

Some studies reported very high visualization rates, around 95% [7, 9, 19–21], but in these cases the authors defined visualization of one of three landmarks (true vocal cord, false vocal cord, or arytenoid cartilage) as success. These authors reported that visualization rate of false vocal fold was much higher than that of true vocal cord. The arytenoid cartilage is one of the landmarks for assessing vocal cord movement; however, it is difficult to generate a clear ultrasonographic image of the arytenoid cartilage using the middle transverse procedure because of its deep position.

Woo et al. [22] described the lateral approach for assessment of vocal cord movement. This procedure, which involves direct transverse observation of the vocal cord, had a very high visualization rate. However, it is difficult to create a good view of the transverse vocal cord and requires mastery of the technique. Furthermore, the vocal cords shift in a cranial direction during phonation, making it difficult to observe the vocal cords transversely during phonation.

**Fig. 6:**
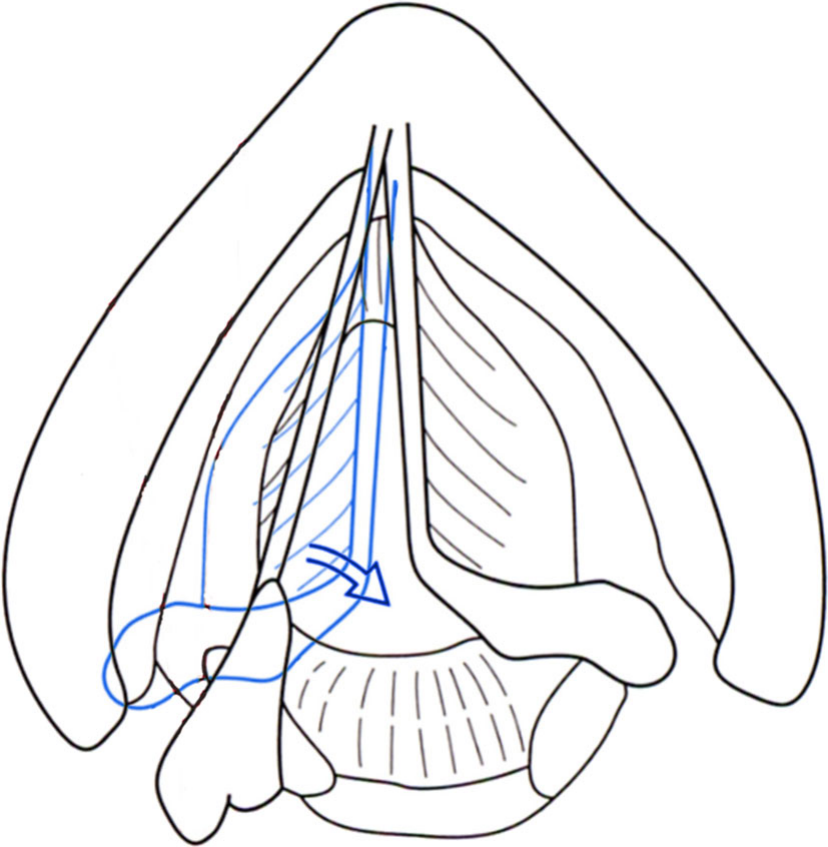
Movement of the arytenoid cartilage during phonation

On the other hand, it was easy to create an image using our lateral vertical procedure for observing movement of the arytenoid cartilage. In the studies cited above, the visualization rate of arytenoid cartilage was high [1–3, 7]. Because the vocal ligament and muscle connect the vocal process and oblong fovea of the arytenoid cartilage, vocal cord movement can be assessed by evaluating movement of the arytenoid cartilage (Fig. 6). When we observe this movement, we can see a dynamic motion in the vertical view because the arytenoid cartilage rotates three-dimensionally just behind the thyroid cartilage. In the surgical procedure (the fenestration approach) for arytenoid adduction, the arytenoid cartilage is accessed through a hole in the lamina of the thyroid cartilage [23, 24]. This surgical procedure demonstrates that the arytenoid cartilage is close to the lamina of thyroid cartilage. In addition, the arytenoid cartilage can be accessed without tearing the pharyngeal mucosa during the procedure. This allows observation of the arytenoid cartilage using a lateral approach and bypassing the air of the laryngopharyngeal space.

We believe that these features explain why the visualization rate of our procedure was superior to that of the conventional middle transverse procedure, and the diagnostic accuracy of our procedure was higher than the conventional procedure.

The lateral approach had its own limitation; namely, the examiners had to perform non-synchronous observation of right and left vocal cord movements. Non-synchronous observation required more time than synchronous observation, and it was difficult to detect minor differences in the vocal cord movement between the right and left sides. Furthermore, in our lateral procedure, we judged vocal cord paresis/paralysis not based on a reduction in movement, but instead based on fixation of the muscular process of the arytenoid cartilage. Consequently, our procedure may be unsuitable for evaluation of vocal cord paresis. However, vocal cord paresis can include a wide range of vocal cord movement: some cases exhibit approximately normal movement, whereas others approximate paralysis. In this study, the only case of vocal cord paresis identified by our lateral vertical procedure was a case of approximate paralysis.

Minor vocal cord paresis is difficult to detect despite the use of nasopharyngoscopy, even for specialists in otolaryngology [25]. Therefore, the need to detect minor vocal paresis by ultrasonography is debatable. However, in cases of vocal cord paresis resulting from thyroid cancer invasion or other causes that require further evaluation, nasopharyngoscopy is the preferred method to evaluate vocal cord movement. Recently developed ultrasonographic systems use a dual-window view, with one window showing a real-time image while the other window plays a recorded movie. Such a system enables comparison of vocal cord movements on the right and left sides in a synchronous manner.

Our lateral vertical procedure is a logical method for approaching inner larynx and improved the visualization rate of vocal cord movement by ultrasonography. We conclude that our novel procedure is useful for ultrasonographic assessment of vocal cord movement, which could be used to screen for vocal cord paralysis.

## Electronic supplementary material

Below is the link to the electronic supplementary material.

Supplementary material 1 (MOV 2368 kb)
